# Sea Anemone (Cnidaria, Anthozoa, Actiniaria) Toxins: An Overview

**DOI:** 10.3390/md10081812

**Published:** 2012-08-22

**Authors:** Bárbara Frazão, Vitor Vasconcelos, Agostinho Antunes

**Affiliations:** 1 CIMAR/CIIMAR, Centro Interdisciplinar de Investigação Marinha e Ambiental, Universidade do Porto, Rua dos Bragas 177, 4050-123 Porto, Portugal; Email: bfrazao@ciimar.up.pt (B.F.); vmvascon@fc.up.pt (V.V.); 2 Departamento de Biologia, Faculdade de Ciências, Universidade do Porto, Rua do Campo Alegre, 4169-007 Porto, Portugal

**Keywords:** Cnidaria, sea anemone, phylogeny, toxin, toxin gene

## Abstract

The Cnidaria phylum includes organisms that are among the most venomous animals. The Anthozoa class includes sea anemones, hard corals, soft corals and sea pens. The composition of cnidarian venoms is not known in detail, but they appear to contain a variety of compounds. Currently around 250 of those compounds have been identified (peptides, proteins, enzymes and proteinase inhibitors) and non-proteinaceous substances (purines, quaternary ammonium compounds, biogenic amines and betaines), but very few genes encoding toxins were described and only a few related protein three-dimensional structures are available. Toxins are used for prey acquisition, but also to deter potential predators (with neurotoxicity and cardiotoxicity effects) and even to fight territorial disputes. Cnidaria toxins have been identified on the nematocysts located on the tentacles, acrorhagi and acontia, and in the mucous coat that covers the animal body. Sea anemone toxins comprise mainly proteins and peptides that are cytolytic or neurotoxic with its potency varying with the structure and site of action and are efficient in targeting different animals, such as insects, crustaceans and vertebrates. Sea anemones toxins include voltage-gated Na^+^ and K^+^ channels toxins, acid-sensing ion channel toxins, Cytolysins, toxins with Kunitz-type protease inhibitors activity and toxins with Phospholipase A2 activity. In this review we assessed the phylogentic relationships of sea anemone toxins, characterized such toxins, the genes encoding them and the toxins three-dimensional structures, further providing a state-of-the-art description of the procedures involved in the isolation and purification of bioactive toxins.

## 1. Introduction

Cnidarians are simple animals with radial symmetry that contain two layers of cells, ectoderm and endoderm. Mesoglea, a non-cellular matrix, is present between the two layers. Cnidarians are mostly predators but certain species may also scavenge dead animals or obtain nourishment from intracellular, photosynthetic unicellular algae, named zooxanthellae.

**Figure 1:**
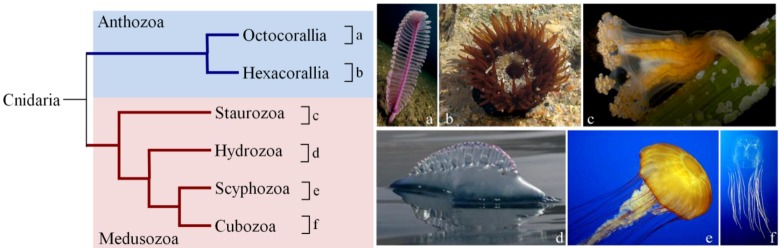
Simplified cladogram of the phylum Cnidaria (adapted from [3]). Photos a, e and f were retrieved from [4].

At least four toxic living classes of cnidarians are currently recognized by most systematists: Anthozoa, Hydrozoa, Scyphozoa and Cubozoa. Molecular phylogenetic methodologies based on DNA sequencing, allowed to determine that the Anthozoa are the basal group of cnidarians [1] (Figure 1). In fact, Anthozoa has a circular mitochondrial DNA, while Hydrozoa, Scyphozoa and Cubozoa have a linear molecule. Likewise the polyp preceded the medusoid form in the course of evolution [2].

**Figure 2:**
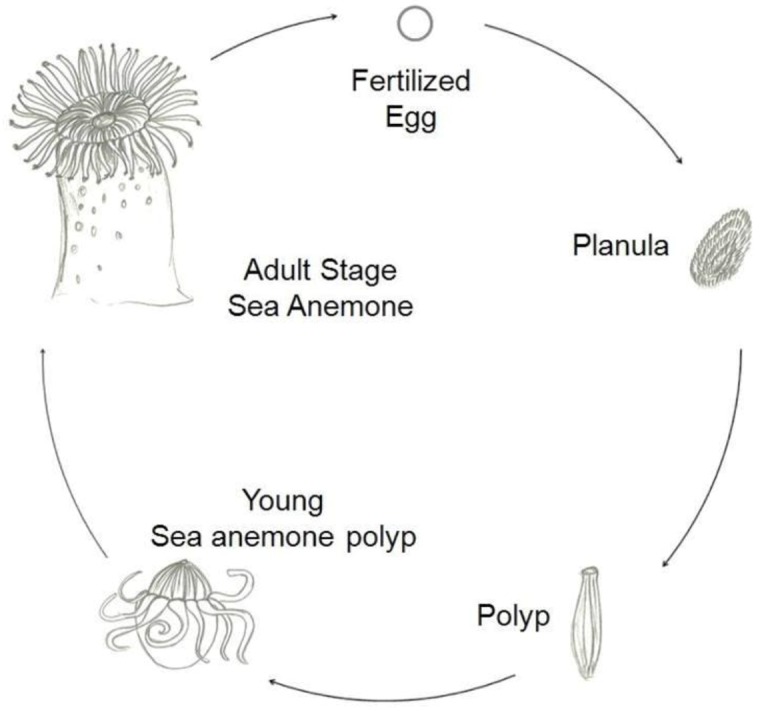
Schematic representation of a typical life cycle of an Anthozoa.

There are two main types of life cycles in cnidarians. In Anthozoans, the polyp is the gamete-producing form and the cycle is embryo > larva > polyp. Medusozoans generally have an embryo > larva > polyp > medusa life cycle, in which the medusa is typically the sexual form. Figure 2 shows a typical life cycle of Anthoza [1]. 

Cnidaria feeding success relies on the presence of specialized poisonous cells, the nematocysts. These organisms have specialized subcellular organelles called cnidae with several structures and functions. Cnidae can be classified into three types: nematocysts, spirocysts, and ptychocysts. Nematocysts deliver the venom through the skin, whereas spirocysts are adhesive and ptychocysts are involved in protection. While Anthozoans have the three types of cnidae, medusozoans (Scyphozoans and Cubozoans) contain only nematocysts. The biological roles of toxins delivered by nematocysts include the capture and killing of prey, digestion, repelling of predators and intraspecies spatial competition [5]. Cnidarians are not just studied by their toxins and venoms, they are a source of marine natural compounds with therapeutically properties, namely antitumor activity [6]. Furthermore, voltage-gated ion channels toxins are studied as an inspiration for drugs design, not only therapeutic but also as insecticides [7].

The composition of cnidarian venoms is not known in detail, but they appear to contain a variety of proteinaceous (peptides, proteins, enzymes and proteinase inhibitors) and non-proteinaceous substances (purines, quaternary ammonium compounds, biogenic amines and betaines) [8]. As an example, palytoxin is a polyether from *Palythoa*, and caissarone is an iminopurine from *Bunodosoma caissarum* [9].

**Figure 3:**
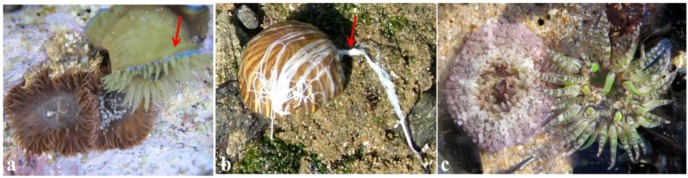
General aspects of the sea anemone morphology. (**a**) Acrorhagi, the blue vesicles in *Actinia equina*, green variety (also called *Actinia prasina*), are used to fight against space towards other individuals (see arrow); (**b**) Acontia, the white threads secreted by *Calliactis parasitica* are used as defensive organs when disturbed (see arrow); (**c**) *Bunodactis verrucosa* specimens with tentacles retracted and fully expanded, illustrating the characteristic column with adhesive verrucae and short tentacles.

The venom is spread all over the body, in a mucous coat, that also protects them from predators, or it is located in the nematocysts. In a recent work, Moran and co-workers, reported that neurotoxin 1 from *Nematostella vectensis* is confined to ectodermal gland cells. Moreover, in *Anthopleura elegantissima* this toxin also appears in gland cells, whereas in *Anemonia viridis* is associated with both nematocytes and ectodermal gland cells [10]. Previously, Honma and co-workers also gave a hint for the same phenomenon when describing that gigantoxins were mostly derived from unknown organelles other than nematocysts [11]. Nematocysts are found mostly on the tentacles, but also exist in other organs such as in acrorhagi and acontia, particularly in certain species of the Actiniidae family, where they are used to fight with nonspecific non-clonemates or for purposes of defence or predation, respectively. Acrorhagi are located in a ring around the base of the tentacles (Figure 3a). Acontia are thin white or color threads attached at one end to the borders of the mesenteries. They can be protruded through the mouth, and in some cases through special pores (cinclides) in the body-wall, for purposes of defence or paralyses of prey (Figure 3b).

The Anthozoa class include sea anemones, and other anemone-like groups with skeletons (such as the “stony” scleractinian corals) and without skeletons (such as tube anemones), as well as sea pens, sea fans, blue corals, and black corals. The word Anthozoa comes from greek *anthos*, flower + *zoon*, animal, as sea anemones resemble flowers (Figure 3c).

Nematocysts possess a high concentration of polypeptides and proteins that act as neurotoxins, hemolysins and enzymes, which are responsible for a variety of harmful effects to humans. These toxins/venoms are only injected in the prey or predator after a mechanical or chemical stimulation [12]. In humans, toxins cause cardiotoxicity, dermatitis, local itching, swelling, erythema, paralysis, pain and necrosis [8]. *In vivo* effects of sea anemone toxins include neurotoxicity and cardiotoxicity.

Summarily, the cnidarians venom includes 3.5–6.5 kDa voltage-gated sodium (Na_V_) channels toxins and 3–5 kDa voltage-gated potassium (K_V_) channel toxins and ~20 kDa pore-forming toxins. The first type prevents inactivation of Na_V_ channels by stabilizing the open state conformations. This fact is due to the binding of the toxin to neurotoxin receptor site 3 [13]. K_V_ channel toxins reversible blocks potassium current and can block acid-sensing ion channels, which are permeable to several cations. The cardiotoxic effects of toxins includes arrhythmias, triggered by early after depolarizations resulting from incomplete Na_V_ channel inactivation, and systolic arrest due to myocardial cell calcium ion overloading [9]. 

Besides toxins, there are several other non-toxic proteins from sea anemones that are studied by its biological activities, such as fluorescent properties [14], but they will not be included in this review. However, we will discuss the importance of protease inhibitors as they adopt a structure that inhibits potassium channels.

In this review, we begin with a brief description of the Anthozoa phylogeny, followed by a general characterization of the sea anemone toxins and afterwards we focus on the major groups of toxins. We then refer to the state of the art techniques used for venom extraction. Afterwards we present the structure of the genes involved in toxin production and the three-dimensional (3D) structures of cnidarian toxins described to date. This review will be solely focused in the molecular diversity of sea anemone toxins. Other cnidarian toxins, as those from coral or jellyfish, will not be considered. More comprehensive information is available in a number of specific papers for jellyfish [15,16], cnidarians in general [2,5,17,18] and sea anemones [13,19,20].

## 2. Phylogenetic Relationships of Anthozoa and Sea Anemone Toxins

Cnidarians are scattered around the world and have around 10,000 estimated species. The majority of the phylogenetic studies classified cnidarians based on morphological characters [21]. At the molecular level, the classification of cnidarians is not yet well established, namely for the order Actiniaria. The phylogeny of Actiniaria is at a suboptimal estimation level [3] and has been retrieved from the sequencing analyses of *12SrRNA*, *16SrRNA*, *18SrRNA*, *28SrRNA* and COIII genes [22,23,24,25]. As referred by Turk and Kem [2], the comprehension of the phylogenetic relationships among Anthozoa members will give insights into the evolution of theirs toxins. Thus, a review about sea anemone toxins could not be dissociated from the Anthozoa phylogenetic characterization.

Besides the few studies on the phylogeny of Actiniaria, some other studies have also been done on the population genetics of these animals. Nonetheless, the majority of those works focus on other Orders, especially on corals. Indeed, few studies were done at the intraspecific level on Actiniaria. Population genetics of *Actinia* spp. assessed with enzyme electrophoresis showed that *Actinia nigropunctata* from Madeira Island (Portugal) is in fact a different species from all the others in the study, as well as *Actinia equina* from Africa [26]. Darling and co-workers in 2006 studied the *Nematostella vectensis* introduced along the Pacific coast of North America and the southeast coast of England, using 10 polymorphic microsatellite loci, and find high variability from Hardy-Weinberg equilibrium as a result of population genetic structure and reproductive plasticity [27].

Considering the molecular markers surveyed in Cnidarians until now, the variation in mitochondrial Citochrome Oxidase I (*COI*), within and between species, is much lower in Anthozoa compared to Medusozoa. Low identification success and substantial overlap between intra- and interspecific *COI* distances render the Anthozoa unsuitable for DNA barcoding [28], with *COI* p-distances among Anthozoa species being equal to 1% [29]. Shearer and co-workers [30] showed that nuclear markers in Anthozoa have much higher substitution rates and therefore should be used instead of mitochondrial genes.

The reduce knowledge on sea anemones phylogeny make it difficult a direct comparison with the toxin genes phylogeny. While previous studies showed a reduced level of congruence between species phylogeny and the toxin gene phylogeny, further research is needed to better clarify this pattern. Such findings may not be unusual due to distinct patterns of toxin gene evolution (e.g., gene duplication/gene loss, horizontal gene transfer, and lineage sorting and diversification). However, future studies are needed to better elucidate the phenomena behind the acquisition and evolution of the toxin genes in Anthozoa.

**Figure 4:**
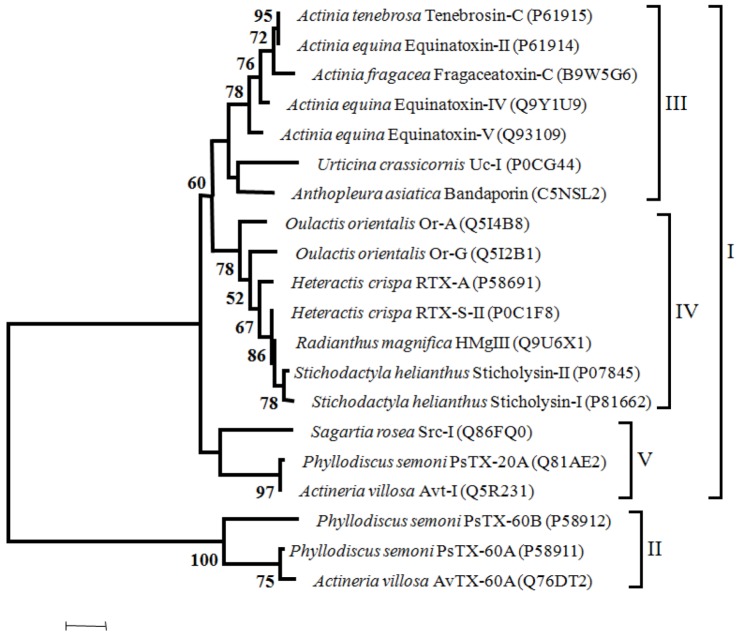
Maximum likelihood tree of Cytolysins with 100 bootstrap replicates (only bootstrap values > 50 are shown). I—proteins without the MACPF domain, II—proteins with the MACPF domain, III—toxins from Actiniidae family members, IV—toxins from Stichodactilidae family members and *Oulactis orientalis* (Actiniidae ), V—toxins from Sagartiidae and and Alisiidae family members. Toxins are also referred on the Cytolysins chapter.

Concerning the phylogeny of toxins, we assessed the phylogenetic relationships of Na_V_ channel and K_V_ channel toxins. In order to systematize the information, we have assessed a phylogenetic tree of cytolysins using only Actinoporins with evidence at transcript level and with full-length sequences. A multiple sequence alignment of amino acids with 533 sites, was made with WebPrank [31] followed by an analysis to choose the best fit model for protein evolution with ProtTest [31], that gave WAG model. A Maximum Likelihood tree reconstruction was made in Mega 5 [32] using 100 bootstrap inferences. A discrete Gamma distribution was used to model evolutionary rate differences among sites (4 categories), Figure 4. (The alignment is available upon request to the corresponding author.)

Considering the phylogenetic tree of cytolysis, two major groups can be defined; one including the proteins without the MACPF domain (I) and the other comprehending those with the MACPF domain (II). Within the major group “I” three clusters can be identified (III to V). Toxins from Actiniidae family members are clustered in group III. In Group IV cluster toxins from Stichodactilidae family members and toxins from *Oulactis orientalis*, (Actiniidae). In fact, toxins from *Oulactis* are more closely related to Stichodactilidae than to Actiniidae toxins. As mentioned previously, Or-A and Or-G and RTX-S-II and RTX-A from *Hecteractis crispa* have in common (albeit others characteristics), the substitution of a Trp by a Leu in the position Trp^112^ of Equinatoxin-II. Moreover, the conserved RGD sequence that occurs in Sticholysin-II, RTX-A and Equinatoxin-II, in the toxins from *Oulactis* is replaced by the GGD sequence. The cluster V includes the Src-I and the toxins from Alisiidae family. The only member of Sagartiidae family (Src-I), has the EGD sequence instead of the RGD motif. Toxins of Alisiidae family members, share a similar gene organization with three exons (two introns). In addition the RGD motif is replaced by the KPS tripeptides in PsTX-20A and Avt. 

Regarding the sea anemones phospholipases toxins, the study of Romero and co-workers [33] comparing PLA2 from *Condylactis gigantea* (Actiniidae family member), CgPLA2, with the other PLA2s from five animal phyla, suggested that sea anemones PLA2s form a monophyletic group. Within this group, CgPLA2 showed to be closer to the *Adamsia carcinoapados* (Hormathiidae family member) PLA2, AcPLA2, than to others of *Nematostella vectensis*, suggesting a significant divergence from the latter.

## 3. General Aspects of Sea Anemone Toxins

**Figure 5:**
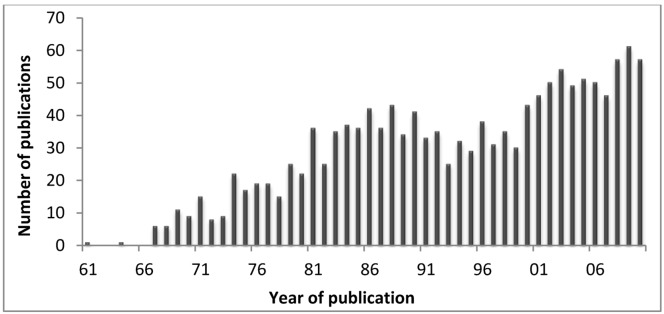
Number of publications from 1961 to date on cnidarians toxins (retrieved from the Pubmed in May 2012).

In the first decades of the 20th century, it was practically impossible to isolate and chemically characterize venom compounds, as the biochemical techniques for isolating such natural products hardly existed [2]. However, nowadays scientists developed several techniques to obtain the venom of particular structures such as acrorhagi or nematocysts, and to separate the venom into fractions. In this sense, there has been an increase in the number of publications on the subject of “cnidarian toxins”. Figure 5 shows the number of publications in Pubmed, retrieved using the query “Cnidaria toxins” on 5 March 2012. In fact, it is expectable that with the deep sequence platforms, much more data will become available at genomic level allowing to better understand the evolution of cnidarians toxins and the discover of the pharmaceutic and therapeutic properties of such compounds. Deep sequencing transcriptomics is the sequencing of the complete set of cellular transcripts at a specific stage or condition, and in that sense Johansen and co-workers [34] and Rodríguez and co-workers [35], pioneered the use of cDNA high-throughput sequencing with 454 pyrosequencing in the discovery of new toxins. The first publications on “cnidarian toxins” were about crude extracts (e.g., jellyfish crude extracts) and not about isolated toxins. In the following years, sea anemones toxins started to gain some relevance. A partial purification of a toxin from the tentacles of *Condylactis gigantea* was made by gel filtration [36]. Afterwards, three neurotoxic peptides were isolated from *Anemonia viridis* by cm-cellulose and sephadex chromatography [37]. The ATX-II amino acid sequence published by Wunderer and co-workers was the first cnidarian toxin to be determined [38]. At same time, in another laboratory, peptides from *Anthopleura* were also studied [2].

Voltage-gated ion channels underlie electrical excitability in cells, and they also play important roles in non-excitable cells. Voltage-gated channels open in response to changes in membrane potential, allowing ions to flow down the electrochemical gradient across the cell membrane, being thus gated (by voltage) and forming an ion-selective pore [17]. Voltage‑gated channels are critical to normal neuromuscular transmission and disruption of their normal function can lead to rapid paralysis. Toxins that target these components [17], are a valuable tool for understanding the structure and function of ion channels [13]. In this review, we will only refer to K_V_ and Na_V_ channel toxins, from all the ion channel toxins known.

Besides neurotoxins, cytolytic sea anemone toxins attracted considerable interest starting in the 1970s. The first report was of a phospholipase A in *Aiptasia pallida* venom [39]. Equinatoxin (Eqt), named following its source, the sea anemone *Actinia equina* [40], was the first actinoporin described in cnidarians.

As referred previously, not all the toxins are related to nematocyst [10,11]. According to Anderluh and co-workers [41], those that are, have a common signal directing them to a maturing cnidocyst [41]. Between the signal peptide and mature region, toxins contain a propart of 9–17 residues long, always ending with Lys-Arg [41]. The propart is composed mainly of polar and negatively charged amino acids, having the role to conduct the toxin to the nematocyst. However, in more recent works it was showed that Nv1 from *Nematostella vectensis* [42] and *Anthopleura elegantissima* toxins have proparts that also end in a Lys-Arg tandem but are not localized in the nematocysts. This suggests that the propart may have another role other than conducting toxins to the nematocysts. In another work, it was found that this cleavage sequence is not always conserved. Indeed, in the AvTX-20 (belonging to Cytolysins Type II) from *Actineria villosa* the propart terminate with a Lys-Lys sequence [43]. 

### 3.1. Na_V_ Channel Toxins

The first representatives of the Na_V_ channel binding proteins were isolated in the 1970s and from all the sea anemone toxins studied, Na_V_ channel toxins are the most thoroughly studied, in part because they constitute a major fraction of the venom [20]. There are four types of these toxic polypeptides of 3.5–6.5 kDa and they bind to the receptor site three of Na_V_ channel during the depolarization procedure. 

Type I and II have 46–51 amino acids and anti-parallel β-sheet with four β-strands and a highly flexible loop, named “Arg-14 loop”, after its most conserved residue, lacking any α-helix [44]. Members of Type I and II have similar locations of the six half-Cys (which form three disulfide bonds), as well as several other residues thought to play a role in biological activity or maintenance of the tertiary structure [19]. In addition, they have basic *C*-terminal sequences [17].

Type III have 27–32 amino acids and rigid β and γ turns. ATX-III and PaTX are representatives of this group and are cross-linked by three and four disulfide bridges, respectively, implying that they do not share the structural scaffold [13]. Moreover, Moran and co-workers studied the bioactive surface of ATX-III and found it consisting mainly of aromatic residues and did not resemble other site-3 toxins, but it also binds the receptor of the site-3 on Na_V_ channels [45]. Type III toxins were identified only in a few species unlike Type I and Type II, which are common in the venom of various cnidarians [17].

Apart from these groups, there is another type of toxins that do not have anything in common with the classic type 3 toxins and therefore are classified as “others”. Calitoxin I and II (79 amino acid residues) resemble Type I and II in the long chain length and in the number of disulfide bridges, three, but not in the amino acid sequence. They act on voltage-gated sodium channels in a similar manner to Type I–III toxins [13]. 

Table 1 indicates all the Na_V_ channel toxins diversity with the described amino acid sequence, theirs accession numbers, their classification group (toxin family), the channel targeted and the Lethal Dose (LD_50_). 

**Table 1:** Sea anemone Na_V_ channel toxins with amino acid sequence described, accession number, their classification group (toxin family), the channel targeted, the LD_50_ and reference.

Species	Toxin	UniProt/GenBank Accession Number	Toxin Family	Target	LD_50_ (µg/kg)/Tested Organism	Ref.
***Actinia equina***	Ae I	Q9NJQ2/AF130344	Type I	-	-	[46]
***Anemonia erythraea***	AETX-I	P69943/-	Type I	-	2.2/Mice	[47]
***Anemonia viridis***	ATX-I	P01533/-	Type I	Na_V_1	-	[48]
***Anemonia viridis***	ATX-II	P01528/-	Type I	Binds to site 3. DmNa_V_, SCN2A and SCN5A	-	[38]
***Anemonia viridis***	ATX-III	P01535/-	Sea anemone short toxin family	Na_V_1	-	[49]
***Anemonia viridis***	ATX-V	P01529/-	Type I	-	-	[50]
***Antheopsis maculata***	Am-3	P69928/AB180687	Type I	-	70/Crabs	[51]
***Anthopleura elegantissima***	Anthopleurin-C	P01532/-	Type I	-	-	[52]
***Anthopleura elegantissima***	APE 1-1	P0C1F0/-	Type I	-	10/Crabs	[53]
***Anthopleura elegantissima***	APE 1-2	P0C1F1/-	Type I	-	-	[53]
***Anthopleura elegantissima***	APE 2-1	P0C1F2/-	Type I	-	1/Crabs	[53]
***Anthopleura elegantissima***	APE 2-2	P0C1F3/-	Type I	-	-	[53]
***Anthopleura fuscoviridis***	AFT-I	P10453/-	Type I	-	-	[54]
***Anthopleura fuscoviridis***	AFT-II	P10454/-	Type I	-	-	[54]
***Anthopleura*** **sp.(strain ** **‘** **Zhanjiang** **’** **)**	Toxin Hk16	P0C5F7/-	Type I	-	-	[55]
***Anthopleura*** **sp.(strain ** **‘** **Zhanjiang** **’** **)**	Toxin Hk2	P0C5F4/-	Type I	-	-	[55]
***Anthopleura*** **sp. (strain ** **‘** **Zhanjiang** **’** **)**	Toxin Hk7	P0C5F5/-	Type I	-	-	[55]
***Anthopleura*** **sp. (strain ** **‘** **Zhanjiang** **’** **)**	Toxin Hk8	P0C5F6/-	Type I	-	-	[55]
***Anthopleura xanthogrammica***	Anthopleurin-A	P01530/-	Type I	Na_V_1	-	[56]
***Anthopleura xanthogrammica***	Anthopleurin-B	P01531/-	Type I	Na_V_1	-	[57]
***Anthopleura xanthogrammica***	Toxin PCR1	P0C5F8/-	Type I	-	-	[58]
***Anthopleura xanthogrammica***	Toxin PCR2	P0C5F9/-	Type I	-	-	[58]
***Anthopleura xanthogrammica***	Toxin PCR3	P0C5G0/-	Type I	-	-	[58]
***Anthopleura xanthogrammica***	Toxin PCR4	P0C5G1/-	Type I	-	-	[58]
***Anthopleura xanthogrammica***	Toxin PCR5	P0C5G2/-	Type I	-	-	[58]
***Anthopleura xanthogrammica***	Toxin PCR6	P0C5G3/-	Type I	-	-	[58]
***Anthopleura xanthogrammica***	Toxin PCR7	P0C5G4/-	Type I	-	-	[58]
***Bunodosoma caissarum***	Bc-III	Q7M425/-	Type I	Na_V_1.5	600/Mice	[59]
***Bunodosoma cangicum***	Cangitoxin	P82803/-	Type I	-	-	[60]
***Bunodosoma cangicum***	Cangitoxin-2	P0C7P9/-	Type I	Na_V_1.1/SCN1A, Na_V_1.5/SCN5A and Na_V_1.6/SCN8A	-	[61]
***Bunodosoma cangicum***	Cangitoxin-3	P0C7Q0/-	Type I	SCN1A/Na_V_1.1	-	[61]
***Bunodosoma granulifera***	NeurotoxinBg-2	P0C1F4/-	Type I	Site 3. SCN2A/SCN1B, SCN4A/SCN1B, SCN5A/SCN1B and para/tipE	0.4/Mice	[62]
***Bunodosoma granulifera***	NeurotoxinBg-3	P0C1F5/-	Type I	Site 3. SCN4A/SCN1B, SCN5A/SCN1B, and para/tipE	21/Mice	[62]
***Calliactis parasitica***	Calitoxin 1	P14531/S69403	Others			[63]
***Calliactis parasitica***	Calitoxin 2	P49127/S69399	Others			[64]
***Condylactis gigantea***	CgNa	P0C20/-	Type I	Na_V_1 TX-sensitive Na_V_	1/Crabs	[65]
***Condylactis passiflora*** **(*syn*-*Condylactis gigantea*)**	Cp I	P0CH42/-	Type I	Site 3	-	[66]
***Cryptodendrum adhaesivum***	Ca I	D2KX90/AB512761	Type II	-	20/Crabs	[67]
***Halcurias carlgreni***	Halcurin	P0C5G6/-	Type II	-	5.8/Crabs	[68]
***Heteractis crispa***	Rm1	P30831/-	Type II	-	-	[69]
***Heteractis crispa***	Rm2	P30783/-	Type II	-	-	[70]
***Heteractis crispa***	Rm3	P30832/-	Type II	-	-	[71]
***Heteractis crispa***	Rm4	P30784/-	Type II	-	-	[72]
***Heteractis crispa***	Rm5	P30785/-	Type II	-	-	[72]
***Heteractis crispa***	Toxin Rc-1	P0C5G5/-	Type I	-	-	[73]
***Heterodactyla hemprichi***	Hh x	D2KX91/AB512762	Type II	-	-	[67]
***Nematostella vectensis***	Neurotoxin Nv1-116.25.1	B1NWS4/EU124461	Type II	Binds to site 3 of voltage-gated sodium channels. DmNa_V_1/TipE	(PD_50_) 76 nmol/kg Blowfly larvae	[42]
***Nematostella vectensis***	Neurotoxin Nv1-116.27.1	B1NWS6/EU124463	Type II	Binds to site 3 of voltage-gated sodium channels. DmNa_V_1/TipE	(PD_50_) 76 nmol/kg Blowfly larvae	[42]
***Nematostella vectensis***	Neurotoxin Nv1-116.28.1	B1NWS7/EU124464	Type II	Binds to site 3 of voltage-gated sodium channels. DmNa_V_1/TipE	(PD_50_)76 nmol/kgBlowfly larvae	[42]
***Nematostella vectensis***	Neurotoxin Nv1-116.37.1	B1NWS5/EU124462	Type II	Binds to site 3 of voltage-gated sodium channels. DmNa_V_1/TipE	(PD_50_) 76 nmol/kg Blowfly larvae	[42]
***Nematostella vectensis***	Neurotoxin Nv1-116.39.1	P0CH46/DS469622	Type II	Binds to site 3 of voltage-gated sodium channels. DmNa_V_1/TipE	(PD_50_) 76 nmol/kg Blowfly larvae	[74]
***Nematostella vectensis***	Neurotoxin Nv1-116.40.1	B1NWS8/EU124465	Type II	Binds to site 3 of voltage-gated sodium channels. DmNa_V_1/TipE	(PD_50_) 76 nmol/kg Blowfly larvae	[42]
***Nematostella vectensis***	Neurotoxin Nv1-116.41.1	A7SCE5/ DS469622	Type II	Binds to site 3 of voltage-gated sodium channels. DmNa_V_1/TipE	(PD_50_) 76 nmol/kg Blowfly larvae	[74]
***Nematostella vectensis***	Neurotoxin v1-116.45.1	B1NWR7/EU124454	Type II	Binds to site 3 of voltage-gated sodium channels. DmNa_V_1/TipE	(PD_50_) 76 nmol/kg Blowfly larvae	[42]
***Parasicyonis actinostoloides***	PA-TX	P09949/-	Sea anemone short toxin family	-	-	[75]
***Radianthus paumotensis***	Rp-II	P01534/-	Type II	-	-	[76]
***Radianthus paumotensis***	Rp-III	P08380/-	Type II	-	-	[77]
***Stichodactyla helianthus***	Sh1	P19651/-	Type II	Na_V_1	-	[78]
***Stichodactyla gigantea***	Gigantoxin-2	Q76CA3/AB110012	Type I	-	70/Crabs	[79]
***Stichodactyla gigantea***	Gigantoxin-3	Q76CA0/AB110015	Type II	-	120/Crabs	[79]
***Stichodactyla haddoni***	SHTX-4	B1B5I9/AB362570	Type II	-	93/Crabs	[80]
***Thalassianthus aster***	Ta I	D2KX92/AB512763	Type II		24/Crabs	[67]

### 3.2. K_V_ channel Toxins

K_V_ channel toxins were discovered in the 1990’s. These 3–5 kDa polypeptide toxins can be grouped into four structural classes: Type I with 35–37 amino acid residues and three disulfide bridges; Type II with 58–59 residues and three disulfide bridges; Type III with 41–42 residues and three disulfide bridges; and Type IV with 28 residues and two disulfide bridges. In Table 2, is indicated all K_V_ channel toxins with the amino acid sequence described, and with the same descriptors mentioned previously for Na_V_ channel toxins. Besides the classification in types, it was included the toxin family reference and the channel type targeted. Even within the same structural class, toxins can differ in selectivity for different subtypes of channels. Furthermore, and as it happens with Na_V_ channel toxins, many of the sea anemone K_V_ channel toxins have not yet their channel selectivity fully characterized [18].

Several of the sea anemone toxins were discovered for its ability to inhibit the binding of radiolabelled α-dendrotoxin to synaptosomal membranes. α-Dedrotoxin from the green mamba snake, binds to K_V_1.1, 1.2 and 1.6 subunits [18]. After this approach, several other different toxins were consequently investigated. The first K_V_ channel blockers, were isolated from marine sources were ShK from *Stichodactyla helianthus* and BgK from *Bunodosoma granulifera*, both from Type I. Since the detection of these two toxins, others have been discovered in *Anemonia viridis* in 1995 and 1998 [81,82], *Radianthus magnifica* in 1997 [83], *Actinia equina* in 1998 [84], *Anthopleura elegantissima* in 2003 and 2004 [85,86], *Antheopsis maculata* in 2005 [51], *Anemonia erythraea* in 2006 [87], *Bunodosoma caissarum* in 2006 and 2008 [88,89] and *Stichodactyla haddoni* in 2008 [80].

**Table 2:** Sea anemone K_V_ channel toxins with amino acid sequence described, theirs accession number, their classification group (toxin family), the channel targeted, the LD_50_ and reference.

Species	Toxin	UniProt/GenBank Accession Number	Toxin Family	Target	LD_50_ (µg/kg)/Tested Organism	Ref.
***Actinia equina***	AeK	P81897/-	Type I	K_V_1	-	[84]
***Anemonia viridis***	SA5 II	P10280/-	Cnidaria kunitz-type proteinase inhibitor/Type II	-	-	[90]
***Anemonia viridis***	kalicludin-1	Q9TWG0/-	Cnidaria kunitz-type proteinase inhibitor/Type II	K_V_1.2	-	[81]
***Anemonia viridis***	kalicludin-2	Q9TWF9/-	Cnidaria kunitz-type proteinase inhibitor/Type II	K_V_1.2	-	[81]
***Anemonia viridis***	kalicludin-3	Q9TWF8/-	Cnidaria kunitz-type proteinase inhibitor/Type II	K_V_1.2	-	[81]
***Anemonia viridis***	BDS-I	P11494/-	Cnidaria kunitz-type proteinase inhibitor/Type II	K_V_3.1, 3.2, 3.4	-	[82]
***Anemonia erythraea***	AETX-K	Q0EAE5/AB259113	Type I	K_V_1	-	[87]
***Anemonia viridis***	kaliseptin	Q9TWG1/-	Type I	K_V_1.2	-	[81]
***Anemonia viridis***	BDS-II	P59084/-	Type III	K_V_3.1, 3.2, 3.4	-	[82]
***Antheopsis maculata***	Am-2	P69930/AB180686	Type III	-	(PD_50_) 420/Crabs	[51]
***Anthopleura elegantissima***	APET x1	P61541/-	Type III	KCNH2 (HERG1) KCNH6 (HERG2), KCNH7 (HERG3)	0/Mice	[85]
***Anthopleura elegantissima***	APET x2	P61542/-	Type III	H(+)-gated Na_V_ ASIC3	-	[86]
***Anthopleura*** **aff.* xanthogrammica***	AXPI-I	P81547/-	Cnidaria kunitz-type proteinase inhibitor/Type II	-	-	[91]
***Bunodosoma granulifera***	Bgk	P29186/-	Type I	K_V_1.1, K_V_1.2 K_V_1.3, K_V_1.6 K_V_3.2	-	[92]
***Bunodosoma caissarum***	BcIV	P84919/-	Type III	-	-/Crabs	[88]
***Bunodosoma caissarum***	Bc-V	P86470/-	Type III	-	-	[89]
***Bunodosoma cangicum***	Toxin Bcg III 31.16	P86461/-	Type III	-	-	[89]
***Heteractis crispa***	Analgesic Polypeptide HC1	B2G331/AM933240	Cnidaria kunitz-type proteinase inhibitor/Type II	Polypeptide inhibitor of vanilloid receptor 1 (TRPV1)	-	[93]
***Heteractis crispa***	Kunitz-type Trypsin inhibitor IV	P16344/-	Cnidaria kunitz-type proteinase inhibitor/Type II	-	-	[94]
***Metridium senile***	Metridin	P11495/-	Type I	-	-	[95]
***Radianthus magnifica***	HmK	O16846/U58107	Type I	K_V_1.2	-	[83]
***Stichodactyla helianthus***	SHPI-1	P31713/-	Cnidaria kunitz-type proteinase inhibitor/Type II	-	-	[96]
***Stichodactyla helianthus***	SHPI-2	P81129/-	Cnidaria kunitz-type proteinase inhibitor/Type II	-	-	[97]
***Stichodactyla haddoni***	SHTX-3	B1B5I8/AB362569	Cnidaria kunitz-type proteinase inhibitor/Type II	-	-	[80]
***Stichodactyla helianthus***	ShK	P29187/-	Type I	K_V_1.1, K_V_1.2 K_V_1.3, K_V_1.4 K_V_1.6	-	[98]
***Stichodactyla haddoni***	SHTX-1/SHTX-2	P0C7W7/-	Type IV	-	430/Crabs	[80]

Type I toxins interfere with binding of radiolabelled dendrotoxin to synaptosomal membranes and block currents through channels with various K_V_1 subunits and also intermediate conductance K(Ca) channels. The residues Ser^20^, Lys^25^ and Tyr^23^, are responsible for the binding of ShK to the rat brain K_V_ channels [13]. Corresponding residues conserved in other toxins are also responsible for the same binding process. The dyad Lys-Tyr is thus considered to be essential for the binding of toxins to K_V_ channels. In fact, scorpion toxins that block K_V_1 channels, have the similar dyad, with the same function [13]. 

Type II toxins, are homologous to Kunitz-type inhibitors of serine proteases. Sea anemone protease inhibitors have been considered to function by inhibiting endogenous proteases in animals themselves or to protect the toxins injected into prey animals or predators from rapid degradation. However, the finding of potassium channel toxins with protease inhibitory activity, such as kalicludines, leads to assume that sea anemone protease inhibitors serve not only as defensive substances but also as offensive substances to paralyze prey animals [13]. Thus, Kunitz-type protease inhibitor toxins, besides serine protease inhibition, also block various types of cation permeating channels, namely the K_V_1.2 channels [99]. 

Type III toxins are not active on K_V_1 subunits. They block currents involving K_V_3 subunits or ERG (ether-a-go-go, K_V_11.1) channels. The human ERG is an essential component of cardiac cells that controls the duration of the plateau phase of the action potential [17]. Type III toxins, such as BDS-I and II, showed to act by modifying channel gating rather than by directly blocking the channel pore. APETx1 blocks the ERG channels [18].

APETx2 is functionally quite unique. Although sharing 36% to 64% sequence identities with Type III K_V_ channel toxins, BDS-I and II and APETx1, it inhibits not potassium channels but acid-sensing ion channels (ASIC3, H^+^-gated Na_V_ channels) in sensory neurons, which are implicated in the modulation of pain sensation. ASICs are formed by homomeric or heteromeric association of six different subunits (ASIC1a, ASIC1b, ASIC2a, ASIC2b, ASIC3, and ASIC4) but only ASIC3 channels and ASIC3-containing channels are affected by APETx2 [13]. This discovery by Diochot and coworkers [86] was very important in the toxinology field. However, more recently [100] it was found that this toxin also affects the voltage-gated sodium channel Na_V_1.8, which raises its value as an analgesic tool, while reducing the value as a specific pharmacological tool, as Na_V_1.8 is also involved in pain-sensing as ASIC3.

Type IV displaces dendrotoxin binding from synaptosomal membranes but their channel blocking specificity is not yet known [98]. 

### 3.3. Cytolysins

Cellular life is dependent on the integrity of cellular membranes that is responsible for controlling the proper transmembrane distribution of solutes. Thus, it is not surprising that membrane permeabilization induced by specifically designed peptides has evolved as a common strategy [101]. 

Several sea anemone species have been reported to produce cytolytic peptides. However, in this review we only refer to the 13 species that have the Cytolysins amino acid sequence described. Cytolysins are important as they serve as model proteins to study protein-lipid membrane interaction [102]. In addition they are also used to study the eradication of tumour cells and parasites [103] and have also cardio-stimulating, dermatonecrotic properties and antihistamine activity [104].

Based on their primary structure and functional properties, Cytolysins have been classified in four polypeptide groups. Type I, consists of 5–8 kDa peptides that form pores in phosphatidylcholine containing membranes and have antihistamine activity. 

Type II, the most numerous toxins within Cytolysins, have been extensible studied and comprise 20 kDa proteins, which are inhibited by sphingomyelin. These type II Cytolysins are also called Actinoporins due to its ability to bind the membrane phospholipids domains of the host organism, oligomerizing and forming cation selective pores [9]. They belong to the unique family of the α-pore-forming toxins (PFTs) [105]. The cations-selective hydrophilic pores of around 1nm cause haemolysis. As referred previously, in contrast with Type II, Type I is not inhibited by sphingomyelin, and are less hemolytical. In fact, Type II toxins have a preference for sphingomyelin containing membranes and are all cysteineless proteins with high isoelectric points (>9.5) [106]. 

Type III toxins have 30–40 kDa and are formed by Cytolysins with or without PLA activity, being only represented to date by the cytolytic proteins from the genus *Urticina* [101].

Type IV toxins are thiol-activated Cytolysins with 80 kDa*. Metridium senile* produces metridiolysin that is so far the only representative of this group of toxins [102].

There is also another group of Cytolysins that have the membrane-attack complex/perforin (MACPF) domain agents. The MACPF family is best studied in the immune system. The membrane-attack complex (MAC) of the complement system and perforin (PF) produced by T-cell and killer cells, form pores of up to 20 nm on the target membrane, which leads to cell lyses and death. PsTX-60A and PsTX-60B from *Phyllodiscus semoni* and AvTX-60A from *Actineria villosa*, from Japanese sea anemones, belong to this group of Cytolysins. Like perforin, these Cytolysins possess an EGF-like domain next to the MACPF domain [101]. These sea anemone toxins were the first report of MACPF proteins in non-mammalian metazoans. Previously, this membrane-attack complex has been also described in bacteria [107]. Furthermore, the presence of these toxins produced by nematocysts was the first reported case of MACPF proteins recruited into venoms. In this sense, the mode of action in the venom might be explained with the pore-forming action in the same way as the MACPF proteins do it in the mammal’s host defence immune system [108]. 

Table 3 summarizes all the sea anemone Cytolysins with amino acid sequence described, and with the same descriptors mentioned for Na_V_ channel toxins.

**Table 3:** Sea anemone Cytolysins, theirs accession no., the cluster that share with them 50% similarity, their classification group (toxin family), the LD_50_ and reference.

Species	Toxin	UniProt/GenBank Accession Number	Toxin family	LD_50_ (µg/Kg)/Tested Organism	Reference
***Actineria villosa***	Avt-I	Q5R231/AB175824	II	-	[109,110]
***Actineria villosa***	Avt-II	D2YZQ3/AB512460	II	-	[111]
***Actineria villosa***	AvTX-60A	Q76DT2/AB107916	MACPF	LDmin <250/Mice	[107]
***Actinia equina***	Equinatoxin-I	P0C1H0/-	II	23/Mice	[112]
***Actinia equina***	Equinatoxin-Ia	P0C1H1/-	II	23/Mice	[112]
***Actinia equina***	Equinatoxin-II	P61914/U41661	II	35/Mice	[112,113]
***Actinia equina***	Equinatoxin-III	P0C1H2/-	II	83/Mice	[112]
***Actinia equina***	Equinatoxin-IV	Q9Y1U9/AF057028	II	-	[114]
***Actinia equina***	Equinatoxin-V	Q93109/U51900	II	-	[115]
***Actinia fragacea***	Fragaceatoxin C	B9W5G6/FM958450	II	-	[116]
***Actinia tenebrosa***	Tenebrosin-A	P30833/-	II	-	[117]
***Actinia tenebrosa***	Tenebrosin-B	P30834/-	II	-	[117]
***Actinia tenebrosa***	Tenebrosin-C	P61915/-	II	-	[118]
***Anthopleura asiatica***	Bandaporin	C5NSL2/AB479475	II	LD100 0.58/Crayfish	
***Heteractis crispa***	Cytolysin RTX-A	P58691/AY855350	I	50/Mice	[119]
***Heteractis crispa***	Cytolysin RTX-S-II	P0C1F8/-	I	70/Mice	[104]
***Oulactis orientalis***	Actinoporin Or-A	Q5I4B8/AY856481	II	-	[120]
***Oulactis orientalis***	Actinoporin Or-G	Q5I2B1/AY861662	II	-	[120]
***Phyllodiscus semoni***	Pstx-20A	Q8IAE2/AB063314	II	50/Shrimp	[43]
***Phyllodiscus semoni***	PsTX-60A	P58911/AB063315	MACPF	-	[121]
***Phyllodiscus semoni***	PsTX-60B	P58912/AB201429	MACPF	-	[121]
***Radianthus magnifica***	HMgI	P58689/-	II	140/Mice	[122]
***Radianthus magnifica***	HMgII	P58690/-	II	320/Mice	[122]
***Radianthus magnifica***	HMgIII	Q9U6X1/AF170706	II	-	[123]
***Radianthus magnifica***	Hemolytic toxin	P39088/-	II	-	[124]
***Sagartia rosea***	Cytolysin Src-I	Q86FQ0/AY247033	II	-	[125]
***Stichodactyla helianthus***	Sticholysin-I	P81662/AJ009931	II	-	[126]
***Stichodactyla helianthus***	Sticholysin-II	P07845/AJ005038	II	-	[126]
***Urticina crassicornis***	Uc-I	P0CG44/-	III	-	[127]
***Urticina crassicornis***	Urticinatoxin	C9EIC7/GQ848199	III	-	[128]
***Urticina piscivora***	Up-1	P0C1G1/-	III	-	[129]

Given the toxins described to date, the production of Cytolysins does not exclude the production of other toxin types, like neurotoxins. Moreover, Cytolysins may have several isoforms—namely five in *Actinia fragacea* [116] and *Actinia equina* [114], three in *Actinia tenebrosa* [117], two in *Oulactis orientalis* [120] and at genomic level, more than 50 different gene sequences have been cloned from *Radianthus magnifica* [130]. In terms of genetic sequence differences there are Actinoporins that are coded by multiple genes that lacks introns, as equinotoxins [114] and Or-A and Or-G, but there are also some genes, namely Avt-I and PsTX-20A, that have 2 introns, 242 bp and around 600 bp long, respectively [131]. 

Regarding the Actinoporins in which the protein 3D structures have been already elucidated, it is worthwhile to mention that there is a conserved putative *N*-terminal amphiphilic α-helix (essential for pore forming activity [43]), a tryptophan-rich stretch (that binds to erythrocyte membranes [131]) and a RGD-motif Arg-Gly-Asp (that provides affinity for certain types of cells [43]), in the primary structure [102]. The conserved RGD sequence/motif, a peculiar property of some Actinoporins, is located on the surface of protein globule nearby POC (phosphocholine) binding site [105]. In this way, the binding of cytolysin to the membranes integrin(s) is made not only by the RGD motif but also by this complementary binding site, the POC. However, not all the Actinoporins share the RGD motif, common to RTX-A, Sticholysin-II and Equinatoxin-II. There are exceptions, with motifs in equal positions but with differences in the amino acid sequence, such as Or-A and Or-G (from *Oulactis*), Src-I (from *Sagartia*), PsTX-20A (from *Phyllodiscus*) and Avt-I (from *Actineria*). They present GGD, EGD and KPS tripeptides, respectively. In the same study, it was found the following differences in functionally regions of *Hecteractis crispa* (RTX-A and RTX-S-II), *Oulactis* (Or-A and G), and some other Actinoporins: (i) Trp is substituted by Leu in the position equivalent to Trp^112^ in the POC binding site of Equinatoxin-II; (ii) 13 and five residues are truncated in N-terminal regions of Or-A and Or-G, respectively [105].

The pore formation produce by Actinoporins, is conducted by a series of steps. First the toxin attaches to the membrane by the specific recognition of sphingomyelin (but neither cholesterol nor phosphatidylcholine) using the aromatic rich region and the adjacent POC binding site. Then the *N*-terminus hydrophobic face is embedding in the lipid-water interface. This is accompanied by extending the *N*-terminus segment, which is oriented in parallel with the membrane and increases the *N*-terminus helicity. Finally, when the toxin oligomerises on the surface of the membrane, the α-helices of three or four monomers insert into the membrane, forming an ion conductive pathway. So, the walls of functional pore consist in α-helices and lipid molecules [105,124]. 

### 3.4. PLA2 Toxins

Phospholipases A2 (PLA2s) catalyze the hydrolysis of 2-acyl ester bonds of 3-*sn*-phospholipids producing fatty acids and lysophospholipids. These enzymes have several important roles in the dietary lipid catabolism, in cell membrane metabolism and inflammatory diseases [33]. They can be associated with the toxicity of several animal groups, such as snakes, insects, mollusks, cnidarians and sponges [128]. PLA2 are presynaptic neurotoxins, blocking nerve terminals by binding to the nerve membrane and hydrolyzing stable membrane lipids. The products of the hydrolysis cannot form bilayers leading to a change in membrane conformation and ultimately blocking the release of neurotransmitters. PLA2 may form dimers or oligomers.

There is a family of secreted PLA2s comprising low molecular weight (13–15 kDa) disulfide-linked proteins that depend on Ca^2+^-ion for enzymatic activity. PLA2s secreted by the pancreas function as digestive enzymes, while others PLA2 are components of venoms. In addition to secreted PLA2s, there are cytosolic Ca^2+^-dependent and independent PLA2-proteins. Based on the molecular structure, PLA2s are classified into various groups numbered from I to XIV and numerous subgroups [5]. Additional types of Phospholipases include phospholipase A1, phospholipase B, phospholipase C, and phospholipase D.

Albeit PLA2s venom properties have been reported for several cnidarians [5], only in a few cases they have been deeply studied. In Table 4, we show all the PLA2 that have the amino acid sequence described.

**Table 4:** Sea anemone PLA2 toxins, accession number and reference.

Species	Toxin	UniProt/GenBank Accession Number	Reference
***Adamsia palliata***	AcPLA2	Q8WS88/AF347072	[132]
***Bunodosoma caissarum***	Cationic protein C1	P0C2M4/-	[8]
***Condylactis gigantea***	Phospholipase A2	D2X8K2/GU046515	[33]
***Urticina crassicornis***	UcPLA2	A7LCJ2/EU003992	[128]

The first cnidarians PLA2 fully sequenced was published in 2002 for *Adamsia carcinoapados*, AcPLA2 [132]. Although AcPLA2 share common features with others PLA2s, such as the *N*-terminal, 12 Cys for putative disulfide formation, and conserved residues found in the sites of activity and Ca^2+^-binding in the catalytically actives PLA2s, it differs in others, lacking two extra Cys (specific structural features of group I) and the *C*-terminal extension (of group II and X). Curiously, it resembles group V PLA2 in respect to the number of Cys and the absence of the *C*-terminal extension, but it does contain a *N*-terminal prepropeptide not found in group V. Additionally, a unique Phe is found in the active site instead of Tyr [132].

The PLA2 from *Bunodosoma caissarum* has a high amino acid sequence identity to the PLA2 group III proteins isolated from the Mexican lizard and the honey bee [8].

UcPLA2 is a PLA2 belonging to group I, isolated from *Urticina crassicornis* inhabiting the northern Pacific Ocean. It is homologous to the AcPLA2, and similar to the Elapidae snake neurotoxic PLAs, suggesting an identical functional role in snake and cnidarians venoms. However, UcPLA2 has some unusual structural features, most notably an Asn at position 27 (instead of a Cys), which is present in the majority of known group I and group II PLA2s. This replacement is rare in invertebrate PLA2s, and has not been found yet in vertebrate toxic and nontoxic PLA2s of group I and group II, with the single exception of the sea lamprey PLA2, which has an Asn at position 27. Also, in UcPLA2 there is a *C*-terminal truncation of six amino acids, including a Cys, so the usual pairing between Cys^27^ and Cys^126^ is not possible. Recently, several similar proteins were also detected in the *Nematostella vectensis*, implying that this type of PLA2 might be more widespread among cnidarians [128].

Recently it was found a PLA2 in *Condylactis gigantea* from Cuba, which is 84% and 61% similar to the *Adamsia carcinoapados* and the *Nematostella vectensis* PLAs, respectively [33].

The toxins from *Condylactis gigantea* and *Adamsia carcinoapados* are more closely related to each other, compared to toxins from *Condilactis* and *Urticina crassicornis*, both belonging to the Actiniidae family, although *Condylactis* and *Adamsia* belong to different superfamilies, Endomyaria (namely Actiniidae family) and Acontiaria (namely Hormathiidae family), respectively. In this sense the phylogeny of the species may not be congruent with its toxins phylogeny, as previously mentioned.

### 3.5. Other Toxins

Besides the toxins described above, some others have not been yet fully characterized, and so the classification types previously referred are not yet known for such toxins.

Apart from these “other toxins” there are others that are classified site-3 sodium channel toxins or K_V_1 potassium channel toxins but are structurally and/or functionally distinct peptides. These include the APETx1 that inhibit an ether-a-go-go related gene potassium channel and the BDS-I and II that show selectivity for K_V_3.4 channels. APETx2 act on acid-sensing ion channels [80].

Until 2005, all the toxins were isolated from the whole body, tentacles or secreted mucus, but Honma and co-workers [13] have been able to isolate toxins from the Acrorhagi, special aggressive organs. These toxins, acrorhagins, have no sequence homologies with other toxins from sea anemones, and a low similarity with toxins from other venomous animals, such as spiders and cone snails. In fact, the low similarities and the location of Cys residues suggest a different conformation [13]. Such differences between acrorhagins and the others toxins suggest that they do not belong to any previously described group of toxins. Bartosz and co-workers [133] implemented a study also in acrorhagi from *Actinia equina* and found that the toxins involved in this conspecific aggression induce tissues necroses by intracellular formation of reactive oxygen species (ROS), being also devoid of paralytic-neurotoxic activity.

AETX II and III toxins, do not have yet known effects produced. However, they are supposedly neurotoxins. They are composed of 59 amino acid residues and have 10 Cys residues, probably forming five disulfide bridges and are very lethal to crabs [13].

Am-I with 27 amino acid residues, differ from all the other toxins by having four Cys residues. Another peculiarity of this toxin is the six copies of the toxin gene in the precursor sequence [13].

Gigantoxin-1 has 35% sequence homology with epidermal growth factors (EGF), and besides EGF activities have also toxic activities. As sea anemones are in the base of the phylogenetic root of the animal kingdom, Honma and co-workers [13] hypothesized that Gigantoxin-1 could be the ancestor of EGFs. 

Table 5 shows the toxins that are not included in the previous types with the same descriptors mentioned for Na_V_ channel toxins but without the toxin type and target. In addition there are features that distinguish them from other toxins.

**Table 5:** Sea anemone toxins not yet included in any previous classification type. Accession number, their classification group (toxin family), the Lethal Dose (LD_50_), the features that distinguish them from the others and the reference.

Species	Toxin	UniProt/GenBank Accession Number	LD_50_ (µg/kg)	Features	Reference
***Actinia equina***	Acrorhagin 1	Q3C258/AB212066	520/Crabs	Produced by acrorhagi	[134]
***Actinia equina***	Acrorhagin 1a	Q3C257/AB212067	-	Produced by acrorhagi	[134]
***Actinia equina***	Acrorhagin 2a	Q3C256/AB212068	80/Crabs	Produced by acrorhagi	[134]
***Actinia equina***	Acrorhagin 2a	Q3C255/AB212069	-	Produced by acrorhagi	[134]
***Actineria villosa***	Avt120	E9RGH6/AB576860	0.085/Mice	Possible similar function as PsTX-115/may inhibit nerve cells	[135]
***Anemonia erythraea***	AETX-II	P69944/-	0.53/Crabs	Toxin Type Not Known. Possible Neurtoxin	[47]
***Anemonia erythraea***	AETX-III	P69945/-	0.28/Crabs	Toxin Type Not Known. Possible Neurtoxin	[47]
***Antheopsis maculata***	Peptide toxin Am-1	P69929/AB180685	830/Crabs	Inhibits ion channels	[51]
***Bunodosoma granulifera***	Granulitoxin	P58305/-	400/Mice	Neurotoxin	[136]
***Phyllodiscus semoni***	Nephrotoxin PsTX-115	P84851/-	-/Rats	Nephrotoxin	[137]
***Stichodactyla haddoni***	EGF-like peptide SHTX-5	B1B5J0/AB362571	-	Has both toxic and EGF activity	[80]
***Stichodactyla gigantea***	Gigantoxin-1	Q76CA1/AB110014	>1000/Crabs	Has both toxic and EGF activity	[79]

## 4. Isolation and Purification of Bioactive Toxins

When scientists first extracted venoms from cnidarians, they started with species that had the more widespread toxic effects known. Thus, medusas have been the first organism studied, and all the subsequent works have used the protocol of Bloom and co-workers in 1998 as the major technique [138]. In that work, jellyfish tentacles were removed, stored in seawater, and vigorously shaken daily and let to settle to allow the release of the nematocysts. To recover nematocysts, the solution was filtered through a fine sieve. Glass beads, sonication or even freeze-thaw cycles were used with subsequent centrifugation of the solution to remove the cell debris from the venom. 

Regarding the venom extraction in sea anemones, many techniques are employed since then and the techniques for extracting the venoms were obviously improved. In general, several techniques allow the extraction of the sea anemones venoms, which can be removed from the entire animal body or just from parts of the body, such as tentacles, acontia or acrorhagi. The tissue can be processed immediately, frozen or freeze-dried. Moreover, the venom can be obtained without animal injury, just by electric stimulation or gently squeezing the sea anemones. The majority of protocols use water to extract the venom, nonetheless there are other solutions that can be used, such as acetone. Most of the works purify the venom after being obtainment, by gel chromatography, followed by reverse phase HPLC. Some of them even go for SDS-PAGE. This chapter does not represent an exhaustive explanation of all the techniques used and improvements since the 1970s but instead provides a brief overview of the most used techniques to make sea anemones venom extractions, so that beginners can have a compilation of them. Table 6, summarizes the most used protocols. It contains the species used in the paper referenced, the tissue type and amount that scientists used, or the number of individuals collected if they have not sacrificed the animal, the technique used for tissue storage, the solvent used for venom extraction, the mechanical treatment used for venom extraction, the technique used for toxin recovery and reference.

**Table 6:** Most employed protocols used for venom extraction in sea anemones. Species used, tissue type, amount of tissue used or number of individuals used, technique used for tissue storage, solvent used for venom extraction, mechanical treatment used for venom extraction, technique used for toxin recovery and reference.

Species	Tissue	Amount	Storage	Solvent	Mechanical treatment	Toxin Recovery	Reference
***Bunodosoma caissarum***	Not damage	10 individuals	Kept alive	Artificial sea water	Electric stimulation	Filtration	[59]
***Heteractis magnifica***	Not damage	1 kg	Kept alive	Water	Stirred (gently)	Filtration, lyophilization	[123]
***Adamsia carciniopados***	Body, Tentacles, Acontia	-	−20 °C	Sodium chloride	Sonication	Centrifugation	[132]
***Stichodactyla gigantea***	Body	5 g	−20 °C	Water	Motor, blender	Centrifugation	[79]
***Heteractis crispa*** ***(syn-Radianthus macrodactylus)***	Body	1 kg	−20 °C	Water, acetone	Bender	Centrifugation, evaporation	[104]
***Aiptasia mutabilis***	Acontia	-	Kept alive and fed	Sodium citrate	Sonication	Centrifugation	[139]
***Bunodosoma caissarum***	Not damage	30 individuals	Kept alive and starved	Artificial sea water	Electric stimulation	Filtration	[140]
***Actineria villosa***	Globular vesicles	-	Kept alive	Phosphate buffer	Shaken (vigorously)	Centrifugation	[109]
***Actinia equina***	Acrorhagi	2 g	Kept alive	Water	Motor, blender	Centrifugation	[134]
***Stichodactyla gigantea***	Tentacles	5 g	Lyophilization	Water	Bender	Centrifugation	[141]
***Condylactis gigantea***	Body	11 kg	−20 °C	Ethanol, acid acetic, acetone	Bender	Filtration, evaporation, centrifugation	[65]
***Anemonia erythraea***	Body	5 g	−80 °C, −20 °C	Water	Motor, blender	Centrifugation	[87]
***Bunodosoma caissarum***	Not damage	-	Kept alive	Artificial sea water	Electric stimulation	Filtration	[88]
***Stichodactyla hadonni***	Body	5 g	−80 °C, −20 °C	Water	Motor, blender	Centrifugation	[80]
***Bunodosoma cangicum***	Not damage	20 individuals	Kept alive	Artificial sea water	Electric stimulation	Filtration	[89]
***Actinia fragacea***	Not damage	50 individuals	Kept alive	Put together in a beaker	Collection of exudate and gently squeezed	Centrifugation	[116]
***Bunodosoma caissarum***	Tentacles	-	Kept alive and starved	Trifluoracetic acid	Freeze-thaw cycles	Centrifugation, filtration	[8]
***Urticina crassicornis***	Not damage	-	Kept alive	Put together in a beaker	Collection of exudate and gently squeezed	Filtration, centrifugation	[128]
***Cryptodendrum adhaesivum, Heterodactyla hemprichii, Thalassianthus aster***	Body	5 g	−80 °C, −20 °C	Water	Motor, blender	Centrifugation	[67]

While there have not been any paper evaluating the technique’s merit, the procedures that do not injury the animal are better for the obvious reason plus the venom seem to be better in terms of purity and it can be achieved also in good amounts. Moreover, frozen the specimens at −20 °C is also practicable, and good results have been obtained even without using −80 °C freezers, so this conservation method would be a good starting point. Using water as a solvent and a blender, followed by centrifugation, is an easy technique not time consuming nor demanding in terms of materials or skills, thus can be also used as a starting point for venom extraction. Regarding the toxins extraction only from the nematocysts, this can be accomplished if subjecting these structures to sonication or freeze-thaw cycles to burst and release the content.

## 5. Toxin Genes

The first toxins to be studied at genomic level were Equinatoxins and they proved to be intronless. Similarly, Or-A and Or-G also do not contain introns [120]. Afterwards it was found that some toxins, namely Cytolysins Avt-I and Pstx-20A, have three exons (two introns). Moreover, they are coded by at least two genes. Such gene arrangement is not exclusive of Cytolysins. The neurotoxic Clx-I and II, and HmK also have genes that are interrupted by two introns and their exon-intron organization is quite similar to the Avt-I genome structure. The introns-exon junctions that are typical donor and acceptor splice sites have followed the GT/AG rule, in which the introns begin with GT and end with AG [131]. In the work of Gendeh and co-workers [142] on HmK, a similar organization on introns-exon junction in scorpion toxins has been reported, suggesting that molecules with similar functions have similar organization at genomic level, therefore implying a common evolutionary path.

More than five equinatoxins genes are found and two isoforms of Equinatoxin-I [114]. ATX-II is encoded by at least seven genes [20]. In 2008, Wang and co-workers [130] showed that magnificalysins (HMgs) are also encoded by a multigene family, with each member encoding an isoform. They cloned more than 50 genes, all intronless. From the *Nematostella vectensis* whole genome release, it was found 13 genes that encode for the Nv1 toxin. Thus, toxins that are encoded by gene families may be more common than previously believed. Indeed, alternative splicing is not commonly assumed for toxins that have not yet the gene(s) sequenced.

The eight genes that code for the same toxin, Nv1, are arranged more or less sequentially and this supports the concerted evolution theory [143]. This theory is corroborated by what it happens in yeast- unequal crossover. In this unusual phenomenon, the sequence of genes is homogenized through unequal crossing over and gene conversion, resulting in an arrangement where two gene family members from one species are more similar to one another, than to their corresponding homologues in other species [143]. The advantage of this mechanism, having several copies of the same gene, is to produce rapidly a huge amount of venom. The nematocyst is discarded after each discharge, and the absence of a specialized venom organ/gland provide emphasis to this hypothesis. They also add that a multigene family, give organisms two more advantages: (i) the rapid transmission of advantageous mutations and (ii) the prevention of the loss of a highly effective toxin [20]. In the same work, some putative toxins in *Anemonia viridis* and *Actinia equina* were found to evolve in the opposite manner, by accelerated evolution, similar to what happens commonly in other venomous animals. Some toxins may escape from the concerted evolution process, diverging rapidly and with mutations being influenced by its selective value and neutral genetic drift [20]. Diversifying selection or Darwinian selection promotes the fixation of non-synonymous substitutions and ‘‘accelerates’’ the diversification of related sequences. This high substitution rate is typical to the region encoding the mature toxin. In contrast, the regions encoding the signal peptide and propart, which are involved in secretion, are usually highly conserved. In fact, in another work, the analysis of ATX-I and ATX-III from *Anemonia viridis*, revealed that besides the differences between the two toxins and 3D structure, the signal was conserved. This has likely been generated by gene fusion and advantageous in transcript stability and intracellular trafficking and secretion [143]. 

More recently, the genes encoding Kunitz-type toxins from *Heteractis crispa* have been studied [144]. Kunitz-type proteins are encoded by four distinct gene families (GS-, RG-, GG-, and GN-gene families). In one family studied (GS), several homologues peptides were found. Moreover, the Open Reading Frame is interrupted by a single intron located at the middle of the signal peptide. The scientists suggest that the gene family in case evolved through gene tandem duplication flowed by adaptive divergence of the reactive site (a particularly group of amino acids). Furthermore, this evolution seem to be lineage-specific, increasing the ability of *Heteractis crispa* to interact with multiple preys and foes [144].

Genes and transcripts of toxins are determined by PCR and degenerate primers by RACE, usually from the cDNA sequence and cloning.

## 6. Three-Dimensional Toxins Structures

Descriptions of sea anemone protein structures involved in venom activity have been determined by nuclear magnetic resonance (NMR) or X-ray crystallography. With the exception of three Actinoporins (Equinatoxin-II, Sticholysin-II and Fragaceatoxin C), all the other toxins were determined by solution NMR. In the same manner, all the toxins have only one chain, except the Actinoporins that have two, or six in the case of fragaceatoxin. Table 7 shows all the sea anemone toxins with three-dimensional structures described, the species from which it was purified, the type, the resolution method, the number of chains of the molecule and the amino acid number, and the RCSB PDB ID.

**Table 7:** Sea anemone toxins with 3D-structures studied. Species where the structure was purified, type, resolution method, number of chains of the molecule and amino acid number, and Pubmed accession number.

Species	Toxin	Type	Method	Number of Chains	Length (amino acid Number)	PDB ID
***Actinia equina***	Equinatoxin-II	Actinoporin	X-Ray Diffraction	2	179	1IAZ
***Actinia fragacea***	Fragaceatoxin C	Actinoporin	X-Ray Diffraction	6	178	3LIM
***Stichodactyla helianthus***	Sticholysin-II	Actinoporin	X-Ray Diffraction	2	175	1GWY
***Anthopleura elegantissima***	APETx1	K_V_ channel	Solution NMR	1	42	1WQK
***Anthopleura elegantissima***	APETx2	K_V_ channel	Solution NMR	1	42	1WXN
***Anemonia viridis***	BDS-1	K_V_ channel	Solution NMR	1	43	1BDS
***Bunodosoma granulifera***	BgK	K_V_ channel	Solution NMR	1	37	1BGK
***Stichodactyla helianthus***	ShK	K_V_ channel	Solution NMR	1	35	1ROO
***Stichodactyla helianthus***	ShPI-1	Kunitz type proteinase inhibitor	Solution NMR	1	55	1SHP
***Anemonia viridis***	ATX-IA	Na_V_ channel	Solution NMR	1	46	1ATX
***Anemonia viridis***	ATX-III	Na_V_ channel	Solution NMR	1	27	1ANS
***Anthopleura xanthogrammica***	Anthopleurin-A	Na_V_ channel	Solution NMR	1	49	1AHL
***Anthopleura xanthogrammica***	Anthopleurin-B	Na_V_ channel	Solution NMR	1	49	1APF
***Condylactis gigantea***	CgNa	Na_V_ channel	Solution NMR	1	47	2H9X
***Stichodactyla helianthus***	Sh1	Na_V_ channel	Solution NMR	1	48	1SH1

The first 3D protein structures of sea anemone were obtained in the 80’s by NMR analysis, for the Anthopleurin-A and ATX-IA [145,146]. However, the first pore-forming toxin to be studied was the Equinatoxin-II in 2001. 

**Figure 6:**
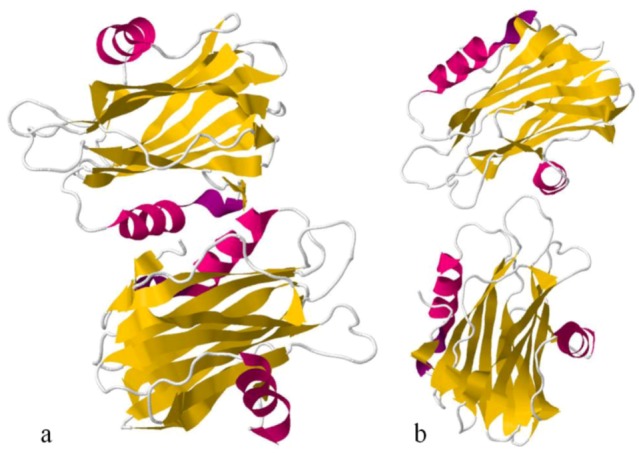
Ribbon view of Equinatoxin-II (**a**) and Sticholysin II(**b**), showing their secondary structure. α-helices, in pink, and β-sandwiches, in yellow.

Sea anemone Actinoporins, Equinatoxin-II and Sticholysin-II, display an extremely similar structural organisation. The molecule is composed of a tightly folded β-sandwich core flanked on two sides by α-helices (Figure 6). The first 30 amino acids encompass one of the helices. This is the only part of the molecule able to undergo a conformational change without any structural change of the β-sandwich. A prominent patch of aromatic amino acids is located on the bottom of the molecule. It comprises a completely exposed Trp^112^ (in EqtII), which was shown to participate in the initial binding of the toxin to the lipid membrane [124]. Comparing Fragaceatoxin C (FraC) with these two other actinoporins, it has a β-sandwich core flanked by three helices and the N-terminal domain is more “detached” from the protein core [147,148].

As previously referred, Actinoporins form pores in membranes by rearranging themselves in a four monomers structure [149]. For this purpose, four regions seem to be important in this step: A cluster of aromatic residues, a phosphocholine binding site, an array of basic amino acids, and the *N*-terminal α-helix. Initial binding of the soluble monomers to the membrane is accomplished by the cluster of aromatic amino acids, the array of basic residues, and the phosphocholine binding site. Then, the *N*-terminal α-helix detaches from the β-sandwich, extends, and lies parallel to the membrane. Simultaneously, oligomerization occurs. Finally, the extended *N*-terminal α-helix penetrates the membrane to build a toroidal pore [150].

**Figure 7:**
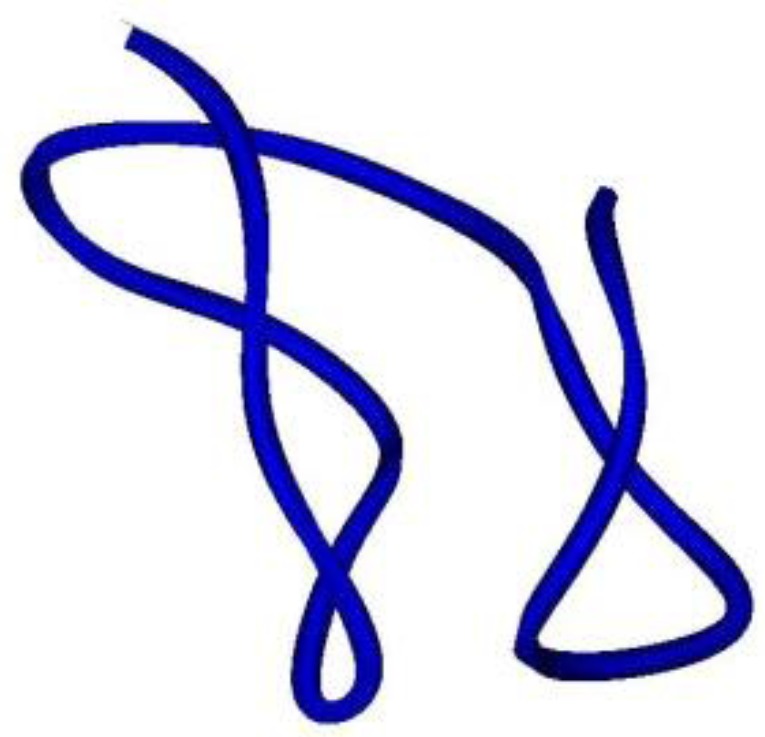
Ribbon view of ATX-III. A peptide toxin from *Anemonia viridis* that interacts with Na_V_ channels. It does not contain α-helixes or β-sheets.

The Na_V_ channel toxin from *Anemonia viridis* has a complete different conformation. ATX III adopts a compact structure, being the smallest of the structures, containing four reverse turns (a distorted type I β-turn, a type I β-turn, and an inverse γ-turn) and two other chain reversals, but no regular α-helix or β-sheet (Figure 7). In this molecule, several of the residues most affected by aggregation are located on the surface of the molecule [151], which cluster on one hemisphere and include a patch of hydrophobic residues only partially exposed [45].

**Figure 8:**
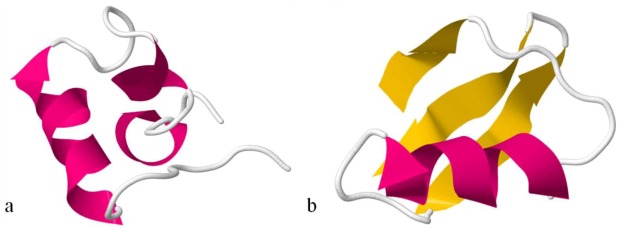
Ribbon structures of BgK(**a**) and ChTx toxins(**b**). BgK lacks the β-sheet secondary structure, while ChTx and most of the scorpion toxins have β-sheet at both ends of the molecule. Also, the molecular scaffolds for the K_V_ channel-binding surfaces of each toxin are of different type: helix (in pink) for BgK and β-sheet (in yellow) for ChTx [153].

**Figure 9:**
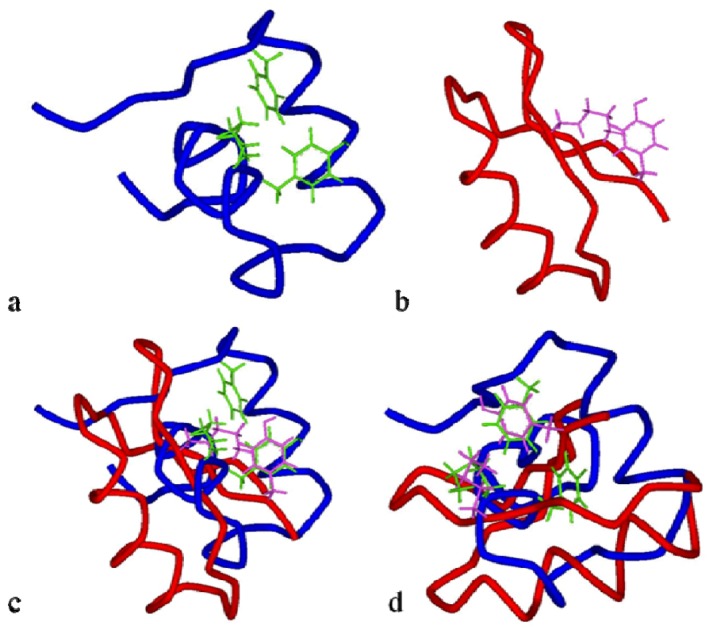
View in tube style of BgK in blue (**a**) and ChTx in red (**b**) molecules, with the residues Lys^25^/Tyr^26^/Phe^6^ in green and Lys^27^/Tyr^36^in pink, respectively. Superimposition of the functional dyad Lys^27^/Tyr^36^ (in pink) from ChTx with the functional dyad of BgK Lys^25^/Tyr^26^ in (**c**) and with the functional dyad Lys^25^/Phe^6^ in (**d**) (adapted from [152]).

With the analysis of the BgK structure, Dauplais and co-workers [152] verified that toxins with different structures from different organisms, like BgK from a sea anemone and ChTX, charybdotoxin (2CRD-PDB Accession Number), from a scorpion, which bind the K_V_ channel, have conserved a dyad. Such a dyad is composed of an essential Lys assisted by a more or less distant aromatic residue, whose precise nature (Tyr or Phe) and location may differ from one toxin to another. This fact suggests a convergent functional evolution for these small proteins [152]. See Figure 8 for structural differences between BgK and ChTx toxins and Figure 9 for conserved dyad between the two toxins.

**Figure 10:**
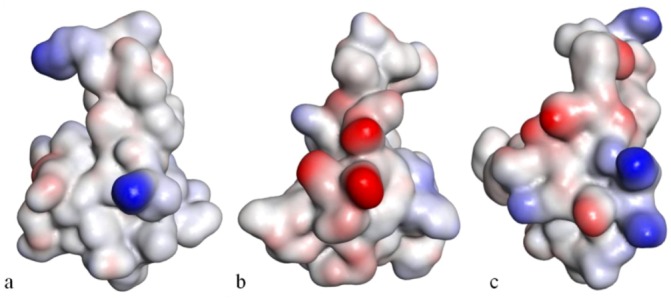
Solvent-accessibility surface representations of Anthopleurin-B (**a**), CgNa (**b**) and Sh1 (**c**), evidencing the electrostatic potential at the surface of the molecule. The color blue represents highly positive and red, highly negative, in grade (adapted from [154]).

The structure of CgNa, which was solved by NMR spectroscopy, is somewhat atypical and display significant homology with both type I and type II anemone toxins in amino acid sequence. CgNa also displays a considerable number of exceptions to the canonical structural elements that are thought to be essential for the activity of this group of toxins. Furthermore, unique residues, as Asp^36^, Glu^37^ and Glu^43^ in CgNa (instead of Trp^45^ in Anthopleurin-B), define a characteristic structure with strong negatively charged surface patches (Figure 10). These patches disrupt a surface-exposed cluster of hydrophobic residues present in all anemone-derived toxins described to date. CgNa preferentially binds to TTX-S (tetrodotoxin-sensitive) Na_V_ channels in the resting state. The specific structural features of CgNa may explain its weaker inhibitory capacity when compared with the other type I and II anemone toxins [154].

ATX-IA, Anthopleurin-A and B and Sh1 are constituted by four-stranded β-sheets. In the ATX-IA they are connected by two loops and there is an additional flexible loop consisting of 11 residues [146]. In Anthopleurin-A and Sh1 they are connected by three loops [155,156] and in Anthopleurin-B by several β-turns [157]. Anthopleurin-A, B and Sh1 have antiparallel β-sheets. APETx1 and BDS-I have three-stranded anti-parallel β-sheets. In addition, BDS-I has one more mini antiparallel β-sheet at the *N*-terminus. The β-sheet is connected by a long exposed loop [158].

The calculated structure of APETx1 belongs to the disulfide-rich all-β structural family, in which a three-stranded anti-parallel β-sheet is the only secondary structure. APETx1 is the first Ether-a-go-go effector discovered to fold in this way [159]. The hERG (the **h**uman **E**ther-à-go-go-**R**elated **G**ene) is a gene (KCNH2) that codes for a protein known as K_V_11.1 potassium ion channel. This ion channel (sometimes simply denoted as “hERG”) is best known for its contribution to the electrical activity of the heart that coordinates the heart’s beating. 

The structures of the K_V_ channel toxins, as BDS-I, APETx1 and APETx2 are similar to those of the Na_V_ channel toxins such as Anthopleurin-A, but quite different from the ShK/BgK family of K_V_ channel toxins. This evidence clearly shows that sea anemones are capable of using a common structural scaffold to create blockers of distinct targets, e.g. Anthopleurin-A, APETx1 and APETx2 act on Na_V_ channel, hERG and ASIC channels, respectively, while also using different scaffolds (all-β in APETx1 *vs.* all-α in ShK) to block similar channels (hERG and K_V_1, respectively) [19].

## 7. Conclusions

In spite of the recent increasing effort to study cnidarians venoms, much more is yet to be done to characterize these compounds in this diverse group of animals. The venom from each species of cnidarians is supposed to contain around 100 compounds, but not more that 1% is currently known even in the better studied species. Indeed, a recent work described 156 peptide venom compounds in a single species [35]. In this sense, toxins such as Actinoporins or PLA2s could be particularly interesting. First, they are less studied than other toxins in cnidarians and second, concerning PLA2s, they are a wide group of toxins also encountered in other animals, as in the better studied snakes. However, much more effort is also needed to pursue the study of ion channel toxins, which will allow a better understanding, not only of the diversity of those toxins, but also of the function of ion channels.

Another field starting to gain relevance is toxin gene detection. The increase in availability of genome sequences for venomous animals or cnidarians in general, added to the development of deep sequencing technology, will enable in depth study of genes encoding toxins. Such genomic studies will not only shed light on the evolutionary mechanisms influencing venom evolution but also, more broadly, on the genetic processes that underline the evolution of novel functionalities.

Cnidarians have impressive strategies for locomotion, feeding and reproduction. Its detailed study may allow unraveling the key for new medical drugs, as well as better understand the diversification of the molecular novelties of these unique metazoan species. For all such reasons, the study of cnidarians in whatever field is of great importance. From genetic and ecological studies to more applied pharmacological and toxicological assessments, these soft body animals should be a target of future scientific research.
